# The Association of microRNA-145 and microRNA-191 with Therapeutic Response in Patients with Inflammatory Bowel Disease

**DOI:** 10.3390/jcm15186996

**Published:** 2026-09-10

**Authors:** Osman Anil Savaş, Hasan Açik, Taner Kivilcim, Amir Mahdi Akbari, Mehrdad Sheikhvatan

**Affiliations:** 1 Department of General Surgery, Istanbul Okan University, 34959 Istanbul, Turkey; osmananilsavas@gmail.com (O.A.S.); taner.kivilcim@okan.edu.tr (T.K.); 2 Department of Internal Medicine, Istanbul Okan University, 34959 Istanbul, Turkey; hasan.acik@hotmail.com; 3 Medical College, Istanbul Okan University, 34959 Istanbul, Turkey; amirmahdiakbari937@gmail.com; 4 Department of Medical Biology and Genetics, Istanbul Okan University, 34959 Istanbul, Turkey; 5 Department of Medical Genetics, Tehran University of Medical Sciences, Tehran 1419733171, Iran

**Keywords:** inflammatory bowel disease, microRNA, biomarker

## Abstract

**Background**: The discovery of non-invasive biomarkers that can predict the therapeutic response in Inflammatory Bowel Disease (IBD) is of utmost importance in the field of personalized medicine. The present prospective cohort study was designed to investigate the use of circulating levels of plasma microRNA-145 (miR-145) and microRNA-191 (miR-191) in the diagnosis and association with therapeutic response in Crohn’s disease (CD) and ulcerative colitis (UC). **Methods**: The present study was conducted as a retrospective observational cohort study on 183 adult IBD patients, consisting of 96 CD patients and 87 UC patients. Patient data and study parameters were obtained retrospectively from existing clinical records and available laboratory/molecular data collected during routine clinical care. A healthy control group consisting of 92 individuals was included for comparison with the patients with IBD. The circulating levels of miR-145 and miR-191 were assessed in the peripheral blood plasma at baseline using qRT PCR. The clinical response was evaluated at 12 weeks using the CDAI score for CD and the Mayo score for UC. **Results**: In active IBD, there was significant down-regulation of miR-145 and significant up-regulation of miR-191. Responders at 12 weeks had significantly higher levels of baseline miR-145 (2.0-fold, *p* < 0.001) and lower levels of miR-191 (1.8-fold, *p* < 0.001) compared with non-responders. Also, miR-145 negatively correlated with disease activity, and miR-191 positively correlated with acute inflammation markers. ROC curve analysis showed good discriminative values for both miR-145 and miR-191. **Conclusions**: The level of circulating miR-145 and miR-191 correlates with the activity of IBD. Importantly, the baseline level of miR-145 expression is a non-invasive potential biomarker associated with therapeutic response at 12 weeks.

## 1. Introduction

Inflammatory bowel disease (IBD), which includes Crohn’s disease (CD) and ulcerative colitis (UC), is a chronic and relapsing inflammatory condition of the gastrointestinal tract that is characterized by intricate interactions between genetic predisposition, environmental factors, gut microbiota, and immune system dysfunction [1,2]. Although there has been significant progress in the development of new therapies, including the emergence of biologic and small molecules, clinical variability and variability in patient response to pharmacologic therapy are currently significant challenges that hinder the achievement of sustained remission and mucosal healing in IBD [3,4]. Thus, the development of reliable predictive biomarkers that can inform personalized treatment approaches is a pressing need in IBD.

MicroRNAs (miRNAs) are small non-coding RNAs that play a significant role in the post-transcriptional regulation of gene expression and are now being appreciated as important regulators of immune and inflammatory responses [5]. Altered miRNA expression profiles have indeed been demonstrated in the intestinal mucosa and circulation of patients suffering from IBD, indicating their role in the pathogenesis, progression, and treatment of the condition [6,7]. Among these, miR-145 and miR-191 have emerged as important miRNAs that regulate the process of differentiation of epithelial cells, immune responses, and the production of inflammatory cytokines, all of which are important in the context of IBD [8,9,10].

New data suggest that abnormal miR-145 and miR-191 expression may be associated with disease severity and treatment response, indicating that these miRNAs have great promise as non-invasive biomarkers that could be used as part of precision medicine strategies for the treatment of IBD [11,12]. However, the predictive value of these miRNAs regarding pharmacotherapy outcome is still undetermined.

The current research seeks to elucidate the significance of the levels of miR-145 and miR-191 in the prediction of the therapeutic response in IBD patients. This research hopes to contribute to the knowledge of the regulatory mechanisms of miRNAs, which may prove instrumental in the development of effective therapeutic strategies for IBD.

## 2. Materials and Methods

### 2.1. Study Design and Patient Selection

The present study was conducted as a retrospective observational cohort study on 183 adult IBD patients, consisting of 96 CD patients and 87 UC patients. Patient data and study parameters were obtained retrospectively from existing clinical records and available laboratory/molecular data collected during routine clinical care. Patients were recruited from the outpatient gastroenterology clinics of Istanbul Okan University during the period of November 2023 to 2024. The diagnosis of the patients with IBD was made based on a combination of clinical, endoscopic, histopathological, and radiological findings. Inclusion criteria were adult patients with established IBD, defined as being 18 years of age or older, starting or receiving standard pharmacological treatment with conventional agents (aminosalicylates, corticosteroids, thiopurines) and biologic agents (anti-TNF agents or integrin inhibitors). The exclusion criteria were pregnancy, presence of malignancy, infectious colitis, recent major surgery, and so forth.

A healthy control group consisting of 92 individuals was included for comparison with the patients with IBD. Healthy controls were recruited from hospital staff attending the hospital for routine health examinations during the study period. Eligibility criteria included age ≥ 18 years, absence of a previous diagnosis of IBD, absence of active gastrointestinal or systemic inflammatory disease, and no history of malignancy, major chronic disease, or recent major surgery. Individuals receiving medications known to substantially affect systemic inflammatory status were also excluded. The healthy control group included 52 men and 40 women, with a mean age of 37.7 ± 10.6 years. Controls were frequency-matched to the patient population according to age and sex. Before sampling, demographic and relevant clinical information were recorded, and participants underwent clinical assessment/routine laboratory evaluation to confirm their healthy status.

### 2.2. Clinical Assessment and Response Evaluation

Disease activity was assessed at baseline and at the 12-week follow-up using validated disease-specific clinical activity indices. For Crohn’s disease (CD), the Crohn’s Disease Activity Index (CDAI) was used. Clinical remission was defined as a CDAI score < 150, and clinical response was defined as a decrease in CDAI of ≥100 points from baseline. For ulcerative colitis (UC), the full Mayo score (total Mayo score, range 0–12) was used. Clinical response was defined as a decrease in the total Mayo score of ≥3 points and ≥30% from baseline, together with a decrease in the rectal bleeding subscore of ≥1 point, whereas clinical remission was defined as a total Mayo score ≤ 2, with no individual subscore > 1.

For the purposes of this study, the term “and/or” indicated that patients were classified as responders if they fulfilled the predefined clinical response criterion and/or achieved clinical remission at week 12. Thus, achievement of clinical remission was considered sufficient for classification as a responder. Baseline was defined as the clinical assessment performed immediately before initiation of a new treatment or modification of the existing treatment regimen. The 12-week assessment was compared with this baseline assessment to determine clinical response or remission. Patients received heterogeneous pharmacological treatments according to their clinical characteristics and the treating physician’s decision, including conventional therapies (aminosalicylates, corticosteroids, and immunomodulators) and biologic therapies (anti-TNF agents or integrin inhibitors). Therapeutic response was evaluated independently of treatment class using the predefined clinical criteria. Because of the sample size and observational nature of the study, treatment-specific response analyses were not performed. Laboratory data, including CRP and fecal calprotectin, were collected to support the clinical assessment.

### 2.3. Sample Collection and RNA Extraction

Peripheral blood samples (5 mL) were collected in EDTA tubes for baseline and follow-up samples. The samples were centrifuged at 3000 rpm for 10 min to separate the plasma, which was later preserved at −80 °C until further processing. Total RNA, including small RNA, was extracted from the samples using the miRNeasy Serum/Plasma Kit (Qiagen, Hilden, Germany), and the manufacturer’s recommendations were followed. The integrity and quantity of the extracted RNA were assessed by NanoDrop spectrophotometer (NanoDrop Technologies, LLC, Wilmington, DE, USA) and Agilent Bioanalyzer (Agilent Technologies, Inc., Santa Clara, CA, USA). All samples were processed and analyzed using the same laboratory procedures to minimize pre-analytical and analytical variability.

### 2.4. Quantitative Real-Time PCR Analysis

Reverse transcription of the microRNAs was performed using the TaqMan™ MicroRNA Reverse Transcription Kit (Applied Biosystems, Foster City, CA, USA). Quantitative real-time polymerase chain reaction (qRT-PCR) was performed using the StepOnePlus™ Real-Time PCR System (Applied Biosystems) and TaqMan™ MicroRNA Assays specific for miR-145, miR-191, and RNU6B with the Assays ID of 002278, 002299, and 001093, respectively. Each sample was analyzed in technical triplicate, and the mean cycle threshold value was used for subsequent analysis. RNU6B was selected as the endogenous reference for normalization and was used consistently across all samples. However, the stability of RNU6B in circulating plasma was not independently validated in the present cohort. Therefore, the use of RNU6B as the endogenous reference should be considered a methodological limitation of the study. To minimize pre-analytical and analytical variability, all blood samples were collected in EDTA tubes, processed using the same centrifugation protocol, stored at −80 °C, and subjected to identical RNA extraction, reverse-transcription, and qRT-PCR procedures.

### 2.5. Statistical Analysis

All statistical tests were conducted with SPSS version 26.0 (IBM Corporation, Armonk, NY, USA) and GraphPad Prism version 9.0 (GraphPad Software, San Diego, CA, USA). Data are expressed as “mean ± standard deviation” or “median with interquartile range” for continuous variables and differences between responder and non-responder patients were analyzed with Student’s *t* test or Mann–Whitney U test for continuous variables and chi-square test for categorical variables. Spearman’s correlation coefficient was used to evaluate the correlation between miRNA expression and clinical indices. Receiver operating characteristic (ROC) curve analysis was used to evaluate the accuracy of miR-145 and miR-191 for therapeutic response. A *p* value < 0.05 was considered statistically significant.

## 3. Results

### 3.1. Patient Characteristics

In all, 183 patients were recruited for this study, with 96 patients diagnosed with Crohn’s disease (CD) and 87 patients diagnosed with ulcerative colitis (UC). Patients’ ages ranged from 18 to 70 years, with an average age of 38.9 ± 12.4 years. Among them, 54.1% were men, and 45.9% were women. No differences in demographic and clinical parameters at baseline, such as disease duration, and levels of inflammatory markers, were noted between patients with CD and UC (Table 1). There were no differences in age and gender between patients who eventually responded to treatment and those who did not. Regarding the treatment regimen, aminosalicylates consumption was used more in UC group than in CD group. In the CD group, no difference was revealed in disease activity based on the CDAI score between responders and non-responders, but in the UC group and according to the Mayo score, the disease activity was significantly higher in non-responders (Table 2).

### 3.2. Expression of miR-145 and miR-191 in IBD

Quantitative real-time PCR analysis showed characteristic expression profiles for miR-145 and miR-191 in both subtypes. At baseline, the relative levels of miR-145 were found to be significantly downregulated in active IBD patients compared with healthy controls (*p* < 0.001), while miR-191 levels were found to be significantly upregulated (*p* = 0.002). No significant difference in miR-145 and miR-191 levels was found between CD and UC patients (*p* > 0.05), suggesting a common feature in both IBD subtypes.

### 3.3. Association Between miRNA Expression and Therapeutic Response

Out of 96 CD patients, 66 (68.8%) showed a clinical response at 12 weeks, while 56 (64.4%) out of 87 UC patients met the criteria for treatment response. In both groups, increased levels of miR-145 at baseline were found to be associated with a positive therapeutic outcome. Responders showed a 2.0-fold increase in miR-145 levels compared with non-responders (*p* < 0.001). On the other hand, miR-191 levels were found to be inversely related to treatment efficacy. Non-responders showed 1.8-fold increased levels compared with responders (*p* = 0.004) (Table 2).

### 3.4. Correlation with Disease Activity and Inflammatory Markers

Spearman’s correlation test for miR-145 serum levels showed negative correlation with disease activity indices such as CDAI (r = −0.46, *p* < 0.001) and Mayo score (r = −0.42, *p* < 0.001). On the contrary, miR-191 levels had a positive correlation with levels of CRP (r = 0.38, *p* < 0.01) and fecal calprotectin (r = 0.34, *p* = 0.02). This indicates their role in systemic and mucosal inflammation.

### 3.5. Discriminative Accuracy of miR-145 and miR-191

**Figure 1:**
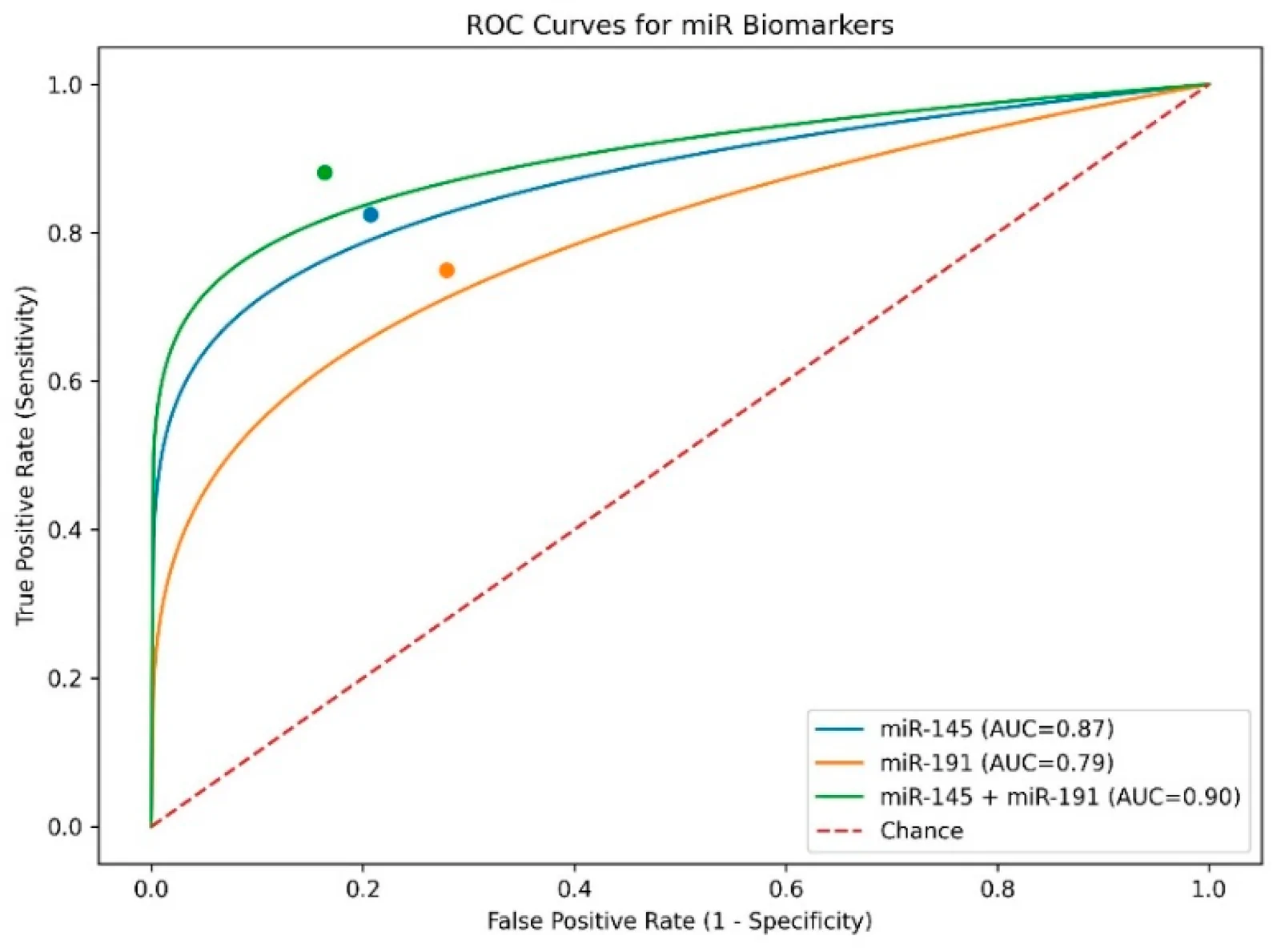
Receiver operating characteristic (ROC) curves for baseline miR-145, miR-191, and their combined predictor for discrimination of clinical treatment response at week 12. The combined predictor was generated using a multivariable logistic regression model incorporating both miRNAs. AUC, area under the curve; CI, confidence interval.

Receiver operating characteristic (ROC) curve analysis was performed to evaluate the ability of baseline miR-145 and miR-191 expression levels to discriminate between patients who achieved clinical treatment response at week 12 and those who did not. miR-145 showed an AUC of 0.87 (95% CI: 0.81–0.92), with an optimal cut-off value corresponding to a sensitivity of 82.5% and a specificity of 79.3%. miR-191 showed an AUC of 0.79 (95% CI: 0.72–0.86), with a sensitivity of 75.2% and a specificity of 70.6% at its optimal cut-off. For the combined analysis, baseline miR-145 and miR-191 levels were entered simultaneously into a multivariable logistic regression model, and the predicted probability of treatment response from this model was used as the combined miR-145/miR-191 predictor. The combined model demonstrated an AUC of 0.90 (95% CI: 0.88–0.93, *p* < 0.001), indicating greater discriminatory ability than either biomarker alone (Figure 1).

## 4. Discussion

The current study set out to evaluate the diagnostic potential of circulating plasma microRNAs, miR-145 and miR-191, in IBD consisting of CD and UC. The findings of this study have established the significance of the baseline expression of these two microRNAs in relation to the therapeutic outcome at 12 weeks.

Our results showed that miR-145 expression levels are downregulated and miR-191 expression levels are upregulated in active IBD patients as opposed to controls. In addition, miR-145 expression levels showed significant negative correlation with established clinical activity indices CDAI and Mayo Score, while miR-191 expression levels showed positive correlation with objective markers of inflammation such as CRP and fecal calprotectin. This is consistent with an emerging literature on miR 145’s role in maintaining gut homeostasis. miR-145 is known to be critical in maintaining epithelial barrier function and differentiation. The down-regulation in active IBD is consistent with findings by Górecka et al. [13] who evaluated circulating serum miRNAs and reported miR-145 as upregulated. On the other hand, the up-regulation of miR-191 is consistent with miR-191’s known role as an oncogenic or pro-proliferative miRNA in various disease states [14]. However, in IBD, the positive correlation with acute inflammatory markers may indicate its release in response to cellular stress or damage, consistent with findings by Chen et al. [15] on circulating microRNAs in acute flare scenarios.

An important finding of the present study was the association between baseline miRNA expression and subsequent clinical response in the pooled IBD cohort. Patients who had achieved clinical response at 12 weeks had significantly higher levels of baseline miR-145 and lower levels of baseline miR-191. The ROC curve analysis also indicated the association of these biomarkers with clinical response with an AUC of 0.87 for miR-145 and a combined AUC of 0.90. However, because Crohn’s disease and ulcerative colitis have distinct clinical characteristics, biological mechanisms, and treatment-response patterns, these findings should be interpreted cautiously. The present analysis does not establish that miR-145 or miR-191 has disease-specific value in either Crohn’s disease or ulcerative colitis. Rather, the findings represent an exploratory association between baseline miRNA expression and overall therapeutic response across the studied IBD population. Additionally, the ROC findings should be interpreted with caution because the discriminatory performance was evaluated in the same cohort from which the associations were derived. No independent validation cohort or internal resampling procedure was available in the present study. Consequently, the observed AUC values, particularly the combined AUC of 0.90, may be subject to optimism and may overestimate the performance that would be observed in an independent population. These findings should therefore be regarded as exploratory, and external validation in independent cohorts, preferably with appropriate internal validation and prespecified models, is required before clinical application can be considered.

The association between higher baseline miR-145 expression and clinical response suggests that miR-145 may have potential value for identifying patients more likely to achieve a favorable overall therapeutic response. However, given the heterogeneity of treatment regimens in the present cohort, these findings should not be interpreted as evidence that miR-145 specifically predicts response to a particular treatment or biologic agent. This is a new finding in terms of its direct application in predicting patient outcomes in a mixed CD/UC patient pool, rather than just diagnosis, as was performed in previous studies. For example, Górecka et al. [13] have previously shown that miR-145 has diagnostic potential in differentiating IBD from healthy controls, but its application in therapy is of greater value.

An important consideration in interpreting these findings is the heterogeneity of treatment regimens in the present cohort. Patients received conventional therapies as well as biologic agents, which differ in their mechanisms of action and expected treatment response. Therefore, the observed associations between baseline miRNA levels and clinical response should be interpreted as reflecting overall therapeutic response rather than response to a specific treatment class. Treatment heterogeneity may have introduced additional variability and could have influenced the observed associations. Future prospective studies with larger sample sizes should evaluate these biomarkers separately according to treatment class and individual therapeutic agents.

The major limitation associated with this research is that it combined CD and UC for the prediction analysis despite the significant difference in baseline subtype distribution between responders and non-responders, as shown in Table 2. Although the molecular signal was significant overall, distinct pathways are involved in CD and UC, and further research should validate these markers for each separately or for each class of biologics. Additionally, although response was measured at week 12, little is known about the durability of response predicted by these markers. Also, an important limitation of this study is the pooling of Crohn’s disease and ulcerative colitis in the primary response analysis. Although both conditions are classified as IBD, they differ in pathophysiology, clinical characteristics, disease activity measures, and therapeutic response. Moreover, the distribution of disease subtype differed between responders and non-responders in our cohort. Therefore, residual disease-subtype heterogeneity may have influenced the observed associations between miRNA expression and therapeutic response. The present findings should consequently be considered exploratory, and larger prospective studies with adequate sample sizes for stratified and adjusted analyses are required to determine whether these associations are consistent within Crohn’s disease and ulcerative colitis separately.

## 5. Conclusions

Circulating miR-145 and miR-191 may have potential value as biomarkers associated with clinical response in patients with IBD. However, given the pooled analysis of Crohn’s disease and ulcerative colitis and the heterogeneity of treatment regimens, these findings should be considered exploratory rather than definitive evidence of disease- or treatment-specific predictive utility. Larger prospective studies with adequately powered stratified analyses are warranted to validate these findings separately in Crohn’s disease and ulcerative colitis. In the revised analysis, the associations between miRNA expression and treatment response were reassessed using the original patient-level dataset. Further prospective and independently validated studies are warranted to confirm the clinical utility of these miRNAs for treatment-response prediction in patients with Crohn’s disease and ulcerative colitis.

## Figures and Tables

**Table 1:** Comparison of baseline demographic and clinical characteristics between CD and UC patients.

Variable	Crohn’s Disease (n = 96)	Ulcerative Colitis (n = 87)	*p*-Value
Age (years)	39.4 ± 12.1	38.3 ± 12.8	0.522
Sex (male/female)	53/43 (55.2%/44.8%)	46/41 (52.9%/47.1%)	0.749
Disease duration (years)	8.1 ± 4.3	7.9 ± 4.6	0.686
Baseline disease activity score	CDAI: 282 ± 72	Mayo: 8.6 ± 2.1	—
C-reactive protein (mg/L)	18.7 ± 11.4	17.9 ± 10.8	0.599
Fecal calprotectin (µg/g)	412.5 ± 173.2	398.3 ± 165.7	0.631
Current medication			
Aminosalicylates	22 (22.9%)	37 (42.5%)	0.010
Corticosteroids	41 (42.7%)	38 (43.7%)	0.912
Immunomodulators (azathioprine/6-MP)	33 (34.4%)	27 (31.0%)	0.644
Biologic therapy (anti-TNF or integrin inhibitor)	28 (29.2%)	24 (27.6%)	0.800
miR-145 relative expression (2^−ΔCt^)	0.64 ± 0.21	0.63 ± 0.18	0.642
miR-191 relative expression (2^−ΔCt^)	1.78 ± 0.43	1.83 ± 0.47	0.473

To compare categorical variables, the Chi-square test was used, and to compare quantitative parameters, the t test was used.

**Table 2:** Comparison of clinical and molecular parameters between therapeutic responders and non-responders (pooled CD and UC cohorts).

Parameter	Responders (n = 122)	Non-Responders (n = 61)	*p*-Value
Sex (Male/Female)	65/57 (53.3%/46.7%)	34/27 (55.7%/44.3%)	0.818
Baseline Disease Subtype (CD/UC)	66/56	30/31	0.536
Baseline Mayo Score (if UC)	7.9 ± 1.9	9.1 ± 1.7	0.007
Baseline CDAI (if CD)	278 ± 65	301 ± 88	0.126
C-reactive protein (mg/L)	14.1 ± 8.5	26.5 ± 13.9	<0.001
Fecal Calprotectin (µg/g)	305.1 ± 105.4	589.6 ± 210.1	<0.001
Baseline miR-145 expression (2^−ΔCt^)	0.85 ± 0.25	0.42 ± 0.19	<0.001
Baseline miR-191 expression (2^−ΔCt^)	1.35 ± 0.31	2.26 ± 0.48	<0.001

To compare categorical variables, the Chi-square test was used, and to compare quantitative parameters, the t test was used.

## Data Availability

The data that support the findings of this study are available from the corresponding author upon reasonable request.
